# KL is a favorable prognostic factor related immune for clear cell renal cell carcinoma

**DOI:** 10.1186/s40001-023-01242-z

**Published:** 2023-09-19

**Authors:** Ke-Hao Pan, Liqing Yao, Zhihao Chen, Jiale Sun, Zongming Jia, Jianglei Zhang, Zhixin Ling

**Affiliations:** 1 Department of Urology, National Cancer Center/National Clinical Research Center for Cancer/Cancer Hospital, Chinese Academy of Medical Sciences and Peking Union Medical College, Beijing, 100021 China; 2 State Key Laboratory of Molecular Oncology, National Cancer Center/National Clinical Research Center for Cancer/Cancer Hospital, Chinese Academy of Medical Sciences and Peking Union Medical College, Beijing, 100021 China; 3 Suzhou Medical College of Soochow University, Suzhou, 215006 China; 4 Department of Urology, The First Affiliated Hospital of Soochow University, 899 Pinghai Road, Suzhou, 215006 Jiangsu China

**Keywords:** Immune, Aging, ccRCC, Prognosis, Biomarkers, KL

## Abstract

**Background:**

Clear cell renal cell carcinoma (ccRCC) is a prevalent cancer in adult urology, often leading to metastasis and poor prognosis. Recently, advances in tumor immunology and aging research have opened up new possibilities for the treatment of kidney cancer. Therefore, the identification of potential targets and prognostic biomarkers for immunotherapy has become increasingly urgent.

**Methods:**

Using GSE168845 data, we identified immune-aging-associated differentially expressed genes (IAR-DEGs) by intersecting differentially expressed immune-related genes and aging-related genes. The prognostic value of IAR-DEGs was determined via univariate and multivariate Cox regression analysis, revealing KL as an independent prognostic factor for ccRCC. We also investigated the correlation between KL and various immune-related factors, including immune cell infiltration, immune score, immune checkpoint, MSI, and TIED score. To confirm the expression of KL in ccRCC, we conducted qRT-PCR assays on both ccRCC cell lines and clinical tissue samples, and compared KL expression levels between normal kidney cell line (HK-2) and ACHN, a ccRCC cell line. Finally, we assessed KL protein expression levels in tissues using immunohistochemistry (IHC).

**Results:**

In this study, we utilized Venn diagram analysis to identify 17 co-expressed immune-aging related DEGs from GSE168845, import database, and MSigDB database. GO and KEGG analysis revealed that the functions of the 17 IAR-DEGs were primarily related to “aging”. Univariate and multivariate Cox analysis validated these 17 genes, and KL was determined to be an independent prognostic factor for ccRCC. The downregulation of KL was observed in ccRCC tissues and was negatively associated with T stage, M stage, pathological stage, and histologic grade (*p* < 0.05). This downregulation indicated disease deterioration and a shortened overall survival period. Our calibration curves and nomogram demonstrated the excellent predictive potential of KL. GSEA analysis showed that KL gene mediated immune and aging-related pathways, and was significantly correlated with immune infiltration and MS and TIED score. More research has revealed a significant reduction in KL mRNA expression levels in human renal cancer cells, particularly in ccRCC tissues compared to adjacent normal kidney tissues. Moreover, immunohistochemistry data have demonstrated a marked decrease in KL protein expression levels in ccRCC cells when compared to adjacent normal tissues.

**Conclusions:**

KL was a potential aging-related target for immunotherapy and valid prognostic biomarker for ccRCC patients.

## Introduction

RCC accounts for about 3% of all cancers, with the highest incidence rate in western countries. Renal cell carcinoma (RCC) is a common malignant tumor of the genitourinary system, accounting for 90% of renal malignant tumors [1]. The incidence rate of RCC has been increasing by 2 to 4% annually since 1975 [2]. RCCs comprise a broad spectrum of histological entities described in the 2016 World Health Organization (WHO) classification [3]. There are three main RCC types: ccRCC (70–80%), pRCC (types I and II, 10–15%, of which 60–70% are type I), and chromophobe RCC (4–5%). Besides the common RCC subtypes described in the 2016 WHO classification, the remaining 10% include renal pelvis carcinoma, collecting duct RCC subtype (malignant epithelial tumors originating from the Bellini collecting duct, high malignant potential with a median survival time of 30 months) and a variety of uncommon, sporadic, and familial carcinomas. Its progression and prognosis are influenced by a complex set of genes [4]. Although surgical resection is effective, 20–38% of patients experience post-surgery progression and metastasis [5]. Therefore, identifying new diagnostic and prognostic biomarkers through studying gene expression changes during ccRCC onset and development is crucial in discovering new therapeutic targets [6]

In 1997, Kuro-o [7] and colleagues first discovered Klotho as a gene related to aging. Later research found that Klotho gene polymorphism was linked to the development and growth of tumors. As a valuable tumor suppressor gene [8], Klotho protein expression is found to be low in various cancerous tissues, including kidney, breast, liver, lung, pancreatic, and others, indicating its ability to inhibit tumor cell growth [9]. A recent study by Zhu et al. [10] demonstrated that Klotho protein expression in renal cell carcinoma is negatively correlated with tumor size and clinical stage.

The expression of Klotho protein has been linked to a higher survival rate in patients with renal cell carcinoma. The activation of cell cycle regulatory proteins p53 and p21 affects the aging of renal tubular epithelial cells. In cultured human umbilical vein endothelial cells, the inhibition of the p53/p21 pathway by Klotho protein counteracts premature aging induced by hydrogen peroxide [11]. Normal renal tubular epithelial cells have high expression of Klotho protein, thus the downregulation of it may impact the p53/p21 pathway and potentially contribute to the development of malignant tumors.

The function and mechanism of klotho in KIRC remain unclear, and further exploration is needed. This paper examines the expression of klotho in normal kidney and KIRC tissues, as well as its prognostic value and correlation with TME. These findings can serve as a basis for KIRC treatment and as a reference for similar research.

## Materials and methods

### Public database collection and gene expression

The TCGA database was used to download the KIRC transcriptome RNA-seq (Workflow Type: HTSeq-counts) and the corresponding patient clinical information, and the data were included until December 2021. The collected data contain RNA-seq information for 537 KIRCs and 71 paracancerous tissues. By the Gene GEO database, the GSE168845 dataset was selected for subsequent analyses. A total of 1793 IRGs (immune-related genes) and and 368 aging-related genes (ARG-MSIGDB) have been downloaded from IMMPORT DATABASE and MSigDB DATABASE.

Differentially expressed genes (DEGs) were screened according to thresholds of |log2foldchange|≥ 1 and *p* < 0.05. Then the intersection was taken with IRGs and ARGs to obtain differentially expressed genes, and the corresponding heat map and volcano map were plotted.

### Functional enrichment analysis

DEGs were subjected to Gene Ontology (GO) and Kyoto Encyclopedia of Genes and Genomes (KEGG) pathway analysis using ClusterProfiler to examine their functional annotation and enriched pathways. Differences were considered significant at *p* < 0.05.

### Survival analysis

We performed univariate and multivariate Cox analyses on 17 IAR-DEGs, with *p* < 0.05 indicating statistical significance. The KL gene was identified as an independent prognostic factor. Prognostic and differential analyses were conducted using the survival R package, with a significant difference defined as *p* < 0.05 and risk ratio (HR) calculated via the Cox proportional hazards and Kaplan–Meier models. The area under curve (AUC) of the time receiver operating characteristic curve (ROC) was used to evaluate the effectiveness of prognostic indicators.

### Construction of clinicopathological correlation analysis and the nomogram

The correlation between the KL gene and clinicopathological characteristics was examined using the “survival” package in R software. To evaluate 1-, 3-, and 5-year OS based on risk scores from prognostic models and clinical traits, we utilized the “rms” package to generate a nomogram and calibration curve.

### GSEA enrichment analysis

In this study, the cluster analyzer package is used, which can efficiently execute GSEA on deg. After that, the software is used to calculate the value, which is less than 0.05, which means it has statistical significance.

### Relationship between KL gene and immune microenvironment

The xCell algorithm of “immune de-noising” is introduced, and the correlation between KL gene and immune cells is discussed in combination with existing relevant literature. In addition, based on the data and information of “ggplot2” R package, the correlation between KL gene and genes related to 8 immune checkpoints was determined. After the above work is completed, TIDE algorithm is introduced to analyze and reveal the immune escape principle of human tumor in combination with KL gene.

### Correlation between microsatellite instability and expression of KL

To further investigate the relationship between microsatellite instability (MSI) and KL, Spearman correlation analysis of MSI and KL gene expression was made.

### Cell lines, patients samples, RNA extraction

The human kidney cell lines HK-2 and human KIRC cell line ACHN used in this study are from the Shanghai Institute of Life Sciences. After collecting the cell sample, put it into 1640 medium (GIBCO) for culture, which contains fetal bovine serum, streptomycin, etc. During this process, it is necessary to maintain 5% carbon dioxide content.

In this paper, a total of 12 fresh samples were selected, and then according to the research needs, the KIRC samples removed by patients in recent years were obtained, and then stored in a high-temperature environment. All patients were diagnosed as KIRC by doctors with rich clinical experience, and all subjects were not given anti-tumor treatment recently. The research in this paper was carried out in accordance with the Helsinki Declaration and has been approved by relevant authorities. All subjects knew the contents and methods of this study, and then signed the informed consent form. Our study is retrospective.

The total RNA kit used in this study was separated according to the manufacturer’s instructions. In addition, the reagent kit (vazyme) is also used. In this paper, the classical 2 − 11Ct method is introduced to complete the normalization of the relevant data, so that the expression of GAPDH can be obtained.

### Tissue microarray construction and immunohistochemistry (IHC)

The samples used in this paper are fixed in formaldehyde solution and then embedded in paraffin. Then process the samples obtained in the above steps with DAB color method. Primary antibody (KL, ab181373, Abcam) in this link.

### Statistical analysis

For the data obtained from the study, the latest version of R software is mainly used for statistics. All indicator data are processed with powerful Perl programming language. For the prognosis, further multivariate Cox regression analysis is needed. Finally, the value of p is calculated. If the value is less than 0.05, it means that the data are significantly different. Fellow researchers can reproduce my experiments through my methods. The number of experiments that we conducted is three times.

## Results

### Identification of IAR-DEGs in KIRC compared to normal renal tissues

**Fig. 1:**
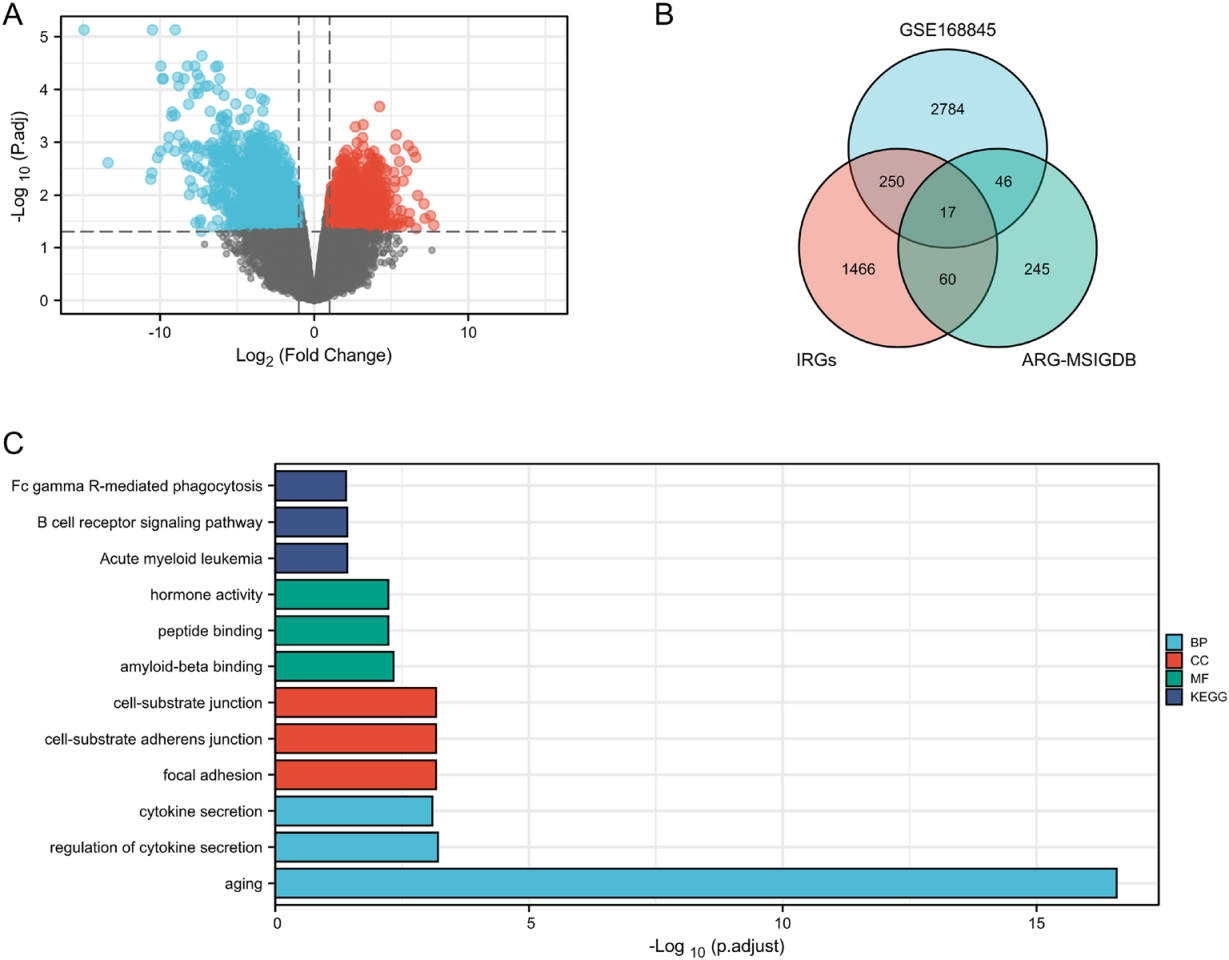
Illustrates the volcano plots of DEGs (differentially expressed genes) between normal renal tissues and renal cancer in GSE168845 samples (**A**). A total of 3097 DEGs were identified, with 1288 being upregulated and 1809 downregulated genes, meeting the criteria of adjusted *p* < 0.05 and log2-fold change (absolute) > 1, The upregulated genes are highlighted in red, while the downregulated genes are depicted in blue. To further investigate immune-related genes (IRGs) and age-related genes (AGs), 1793 human IRGs were downloaded from IMMPORT DATABASE, and 368 human AGs were obtained from Msigdb (https://www.gsea-msigdb.org/gsea/msigdb/index.jsp/). A Venn diagram was generated to show the 17 immune-aging genes shared by the three datasets (**B**). Finally, the GO and KEEG analysis of the 17 immune-aging genes was illustrated in a graph (**C**)

The volcano map visually shows 1288 upregulated DEGs selected by the research team, and 1809 downregulated DEGs, as shown in Fig. 1A. In this link, 1793 human IRGs and and 368 ARGs were also objectively reflected by Venn map, and on this basis, 17 co-expression genes were further identified: TNFRSF1B, GBP2, FCGR2B, LIMSI, INPP5D, PTH1R, CALCA, CAT, LRP1, KL, ARG2, PML, MPO, HAMP, HCST, ITGB2 and MAPK1 (Fig. 1B). In the analysis of GO and KEGG, it turned out that the functions of the 17 co-expressed genes were mainly focused on “aging”, “B cell receptor signaling pathway” and “regulation of cytokine secretion” (Fig. 1C).

### Differential expression analysis and survival analysis of KL gene in KIRC

**Fig. 2:**
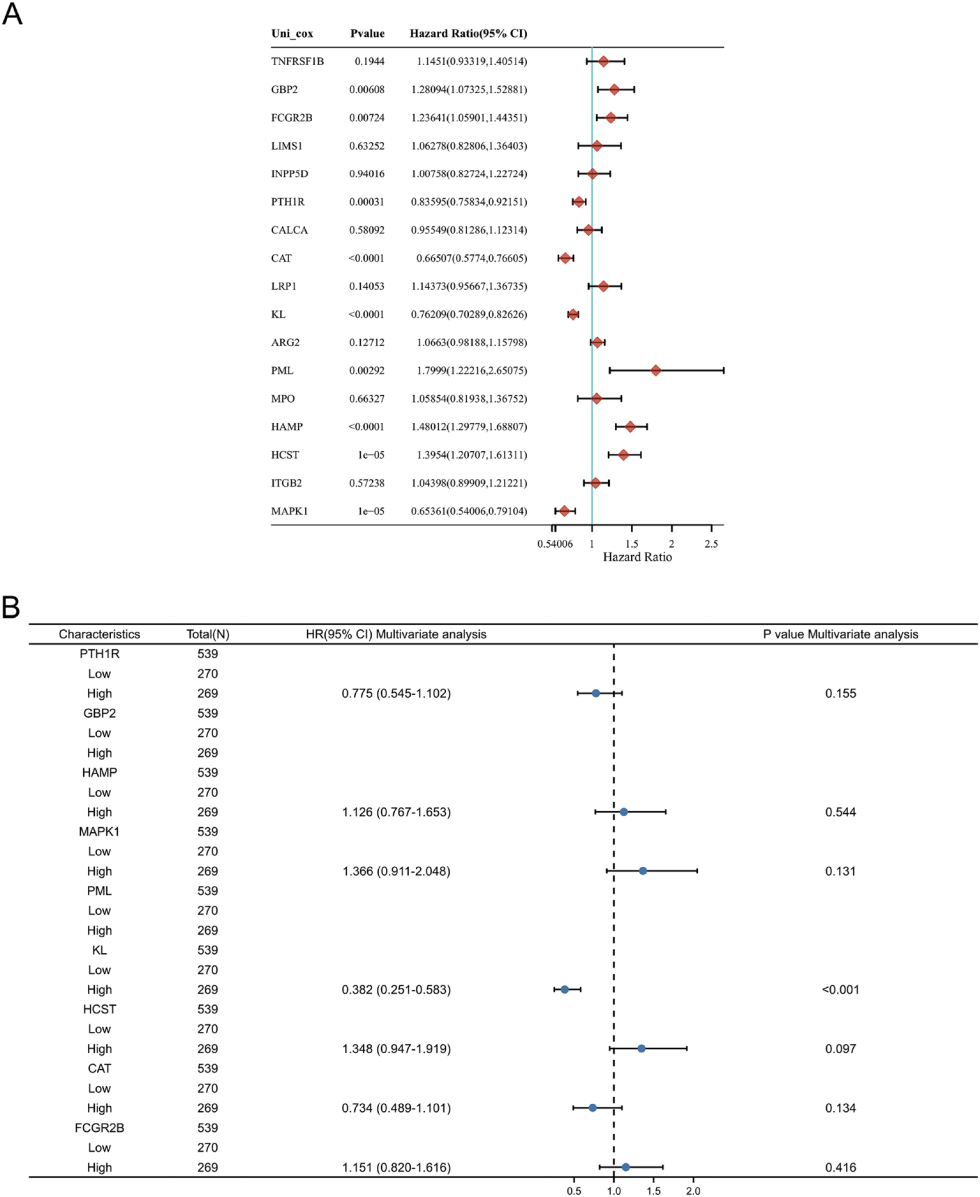
Presents the forest plots displaying the univariate and multivariate Cox regression analyses of the 17 immune-aging genes in TCGA-KIRC (**A** and **B**)

In this paper, single-factor **(**Fig. 2A) and multi-factor (Fig. 2B) Cox regression analysis is introduced to summarize the impact of 17 DEGs on the prognosis. The conclusion is that KL gene can affect KIRC.

**Fig. 3:**
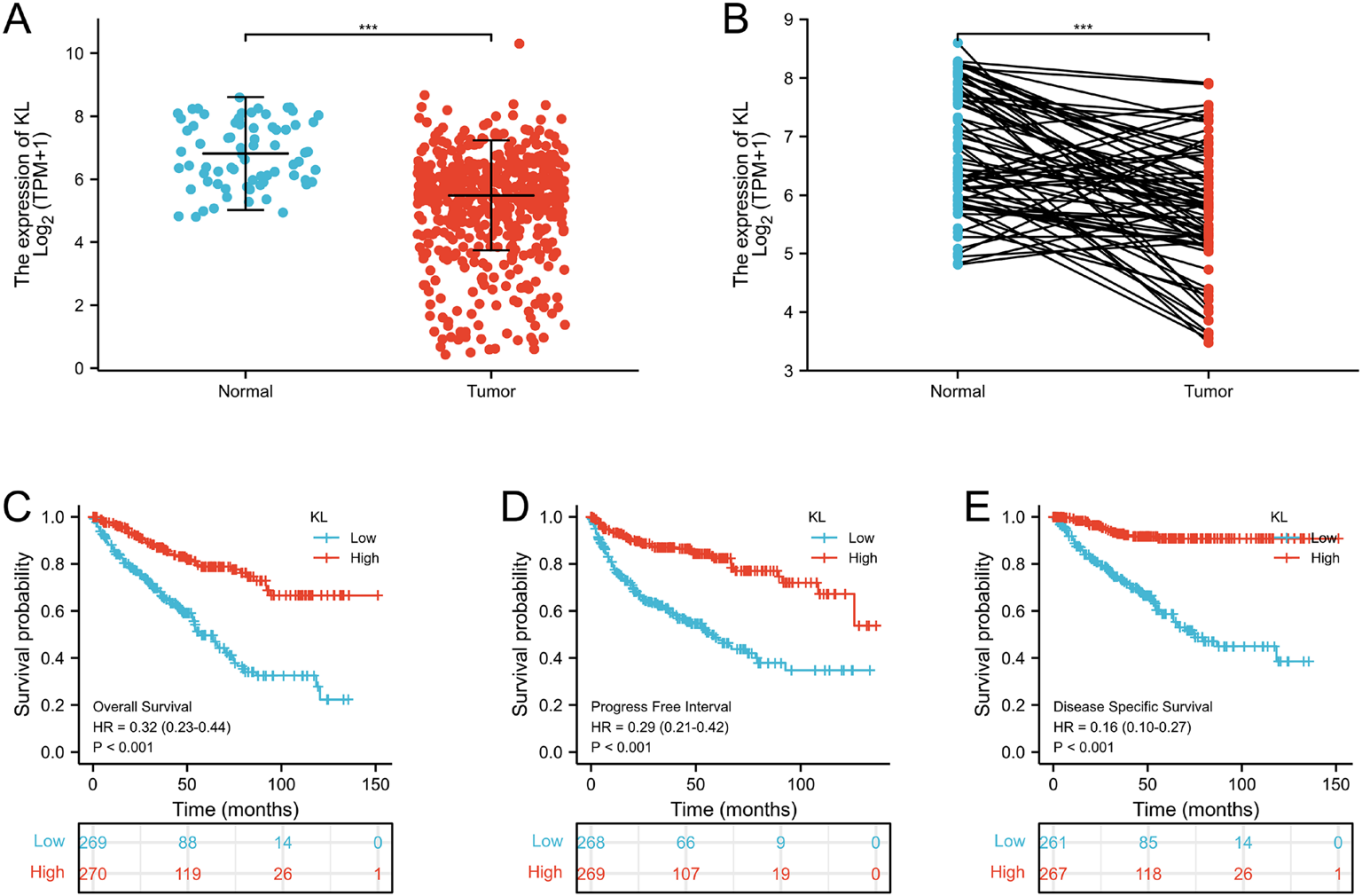
Displays the expression profile of the KL gene in KIRC samples compared to normal tissues (**A**, **B**) and the prognostic value of KL shown through Kaplan–Meier plots (**C**–**E**). Statistical significance is denoted by *p < 0.05, **p < 0.01, and ***p < 0.001

After collecting and processing the data in TCGA-KIRC database, we found KL gene in tumor tissues was downregulated (Fig. 3A, B). Kaplan–Meier model analysis showed that the increased content of KL gene in tissues would reduce the prognosis effect (Fig. 3C–E).

### Clinicopathological correlation analysis and the nomogram construction of risk score

**Fig. 4:**
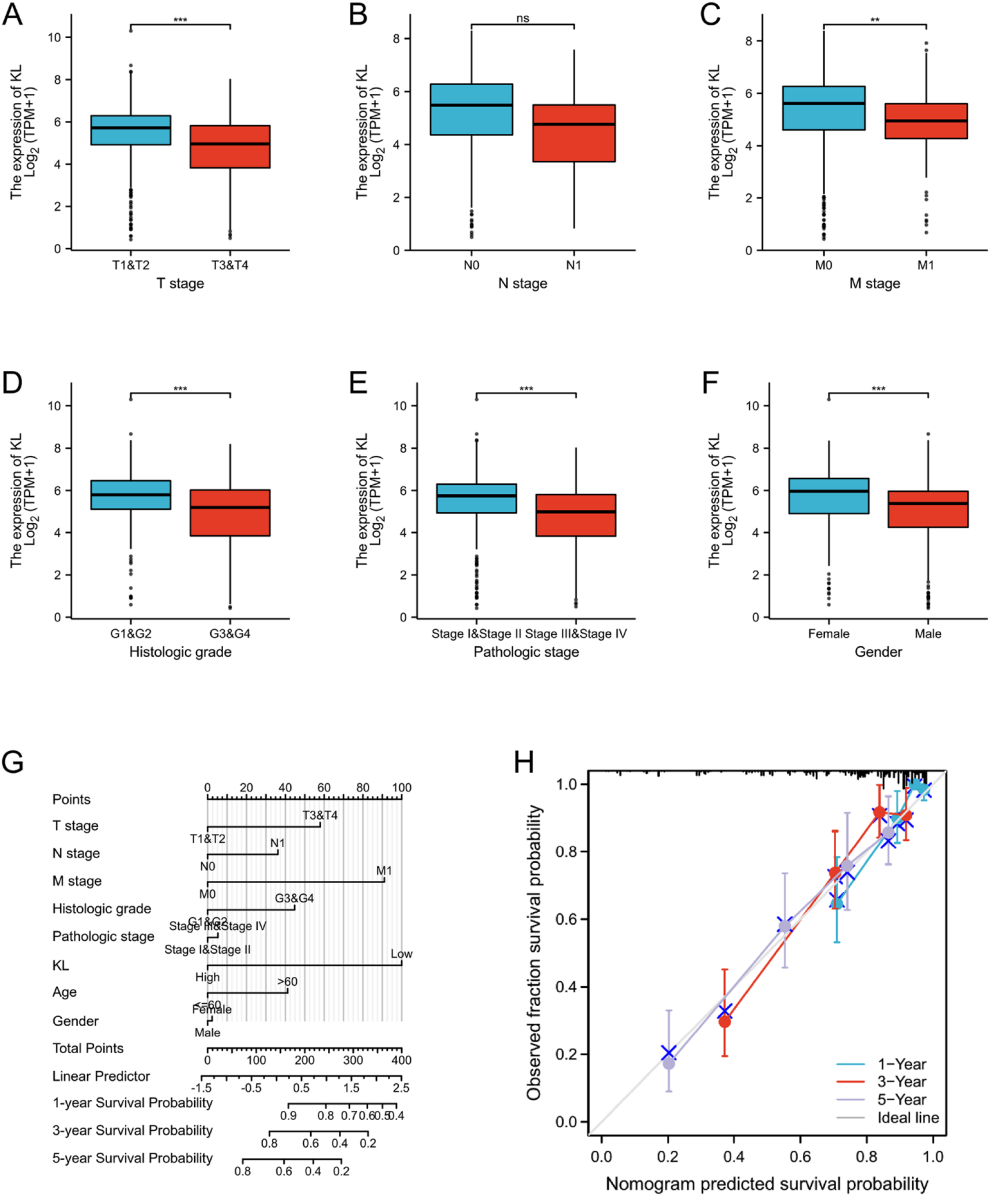
Shows the significant correlation between the KL gene and various clinicopathological factors in KIRC patients. The relationships between KL and clinicopathological factors in the entire TCGA cohort are also presented (**A**–**F**). Additionally, a nomogram for predicting 1-, 3-, and 5-year OS in the entire TCGA cohort is included (**G**), along with calibration curves to measure the consistency between predicted and observed survival rates (**H**). *p < 0.05, **p < 0.01, and ***p < 0.001

We analyzed the correlation between the KL gene and clinicopathological features in KIRC and observed a significant decrease in KL expression between different stages and grades of the disease (Fig. 4A–F). Nomograms were used to predict 1-, 3-, and 5-year OS of KIRC patients and evaluate the correlation between related variables and prognosis (Fig. 4G). We also found that the calibration curves of 3- and 5-year OS based on risk score were consistent with the prediction probability on the nomograms (Fig. 4H). These findings suggest that the risk prognostic model is closely linked to the clinical status and survival time of KIRC patients.

### GSEA analysis of KL gene

**Fig. 5:**
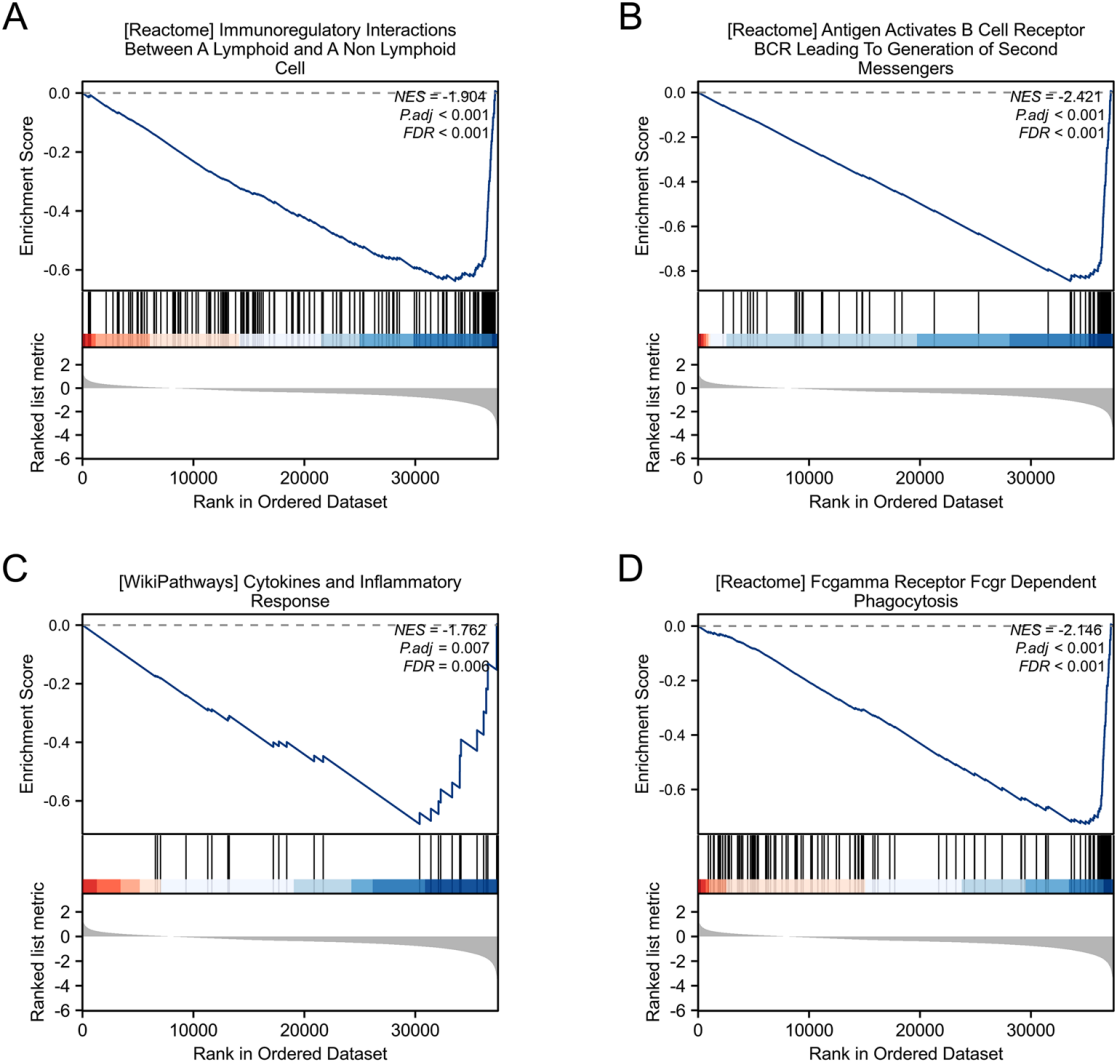
Shows the single gene enrichment analysis of KL, presented in panels **A**–**D**

We used collected a large number of KIRC patient data in the TCGA-KIRC database, and then used GSEA to conduct in-depth analysis of these data. The conclusion is that KL mediated immune and aging-related pathways (Fig. 5A–D).

### Relation between KL gene and immune cells in KIRC

**Fig. 6:**
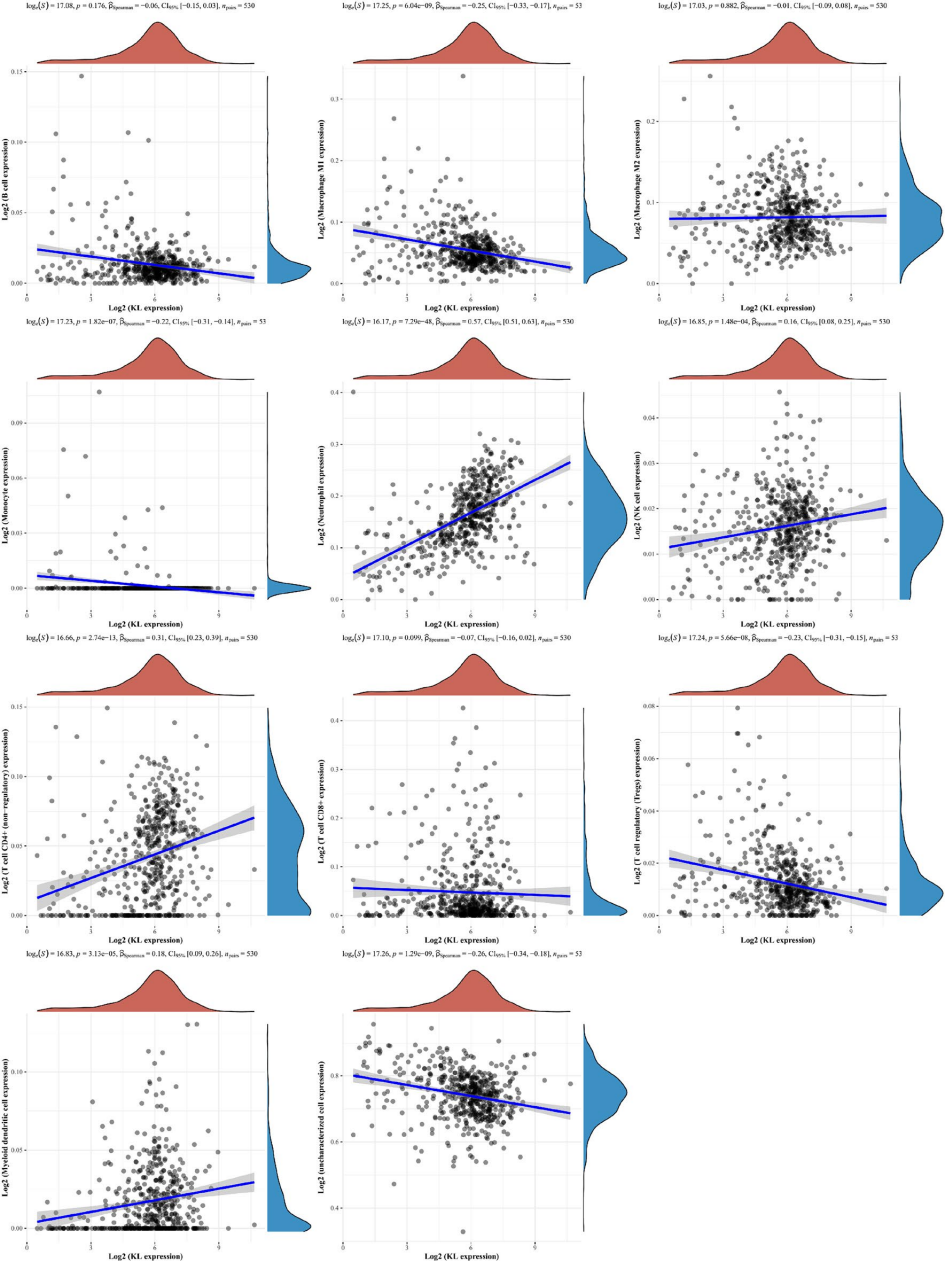
Correlation between KL gene and immune score evaluated using Spearman’s correlation analysis

**Fig. 7:**
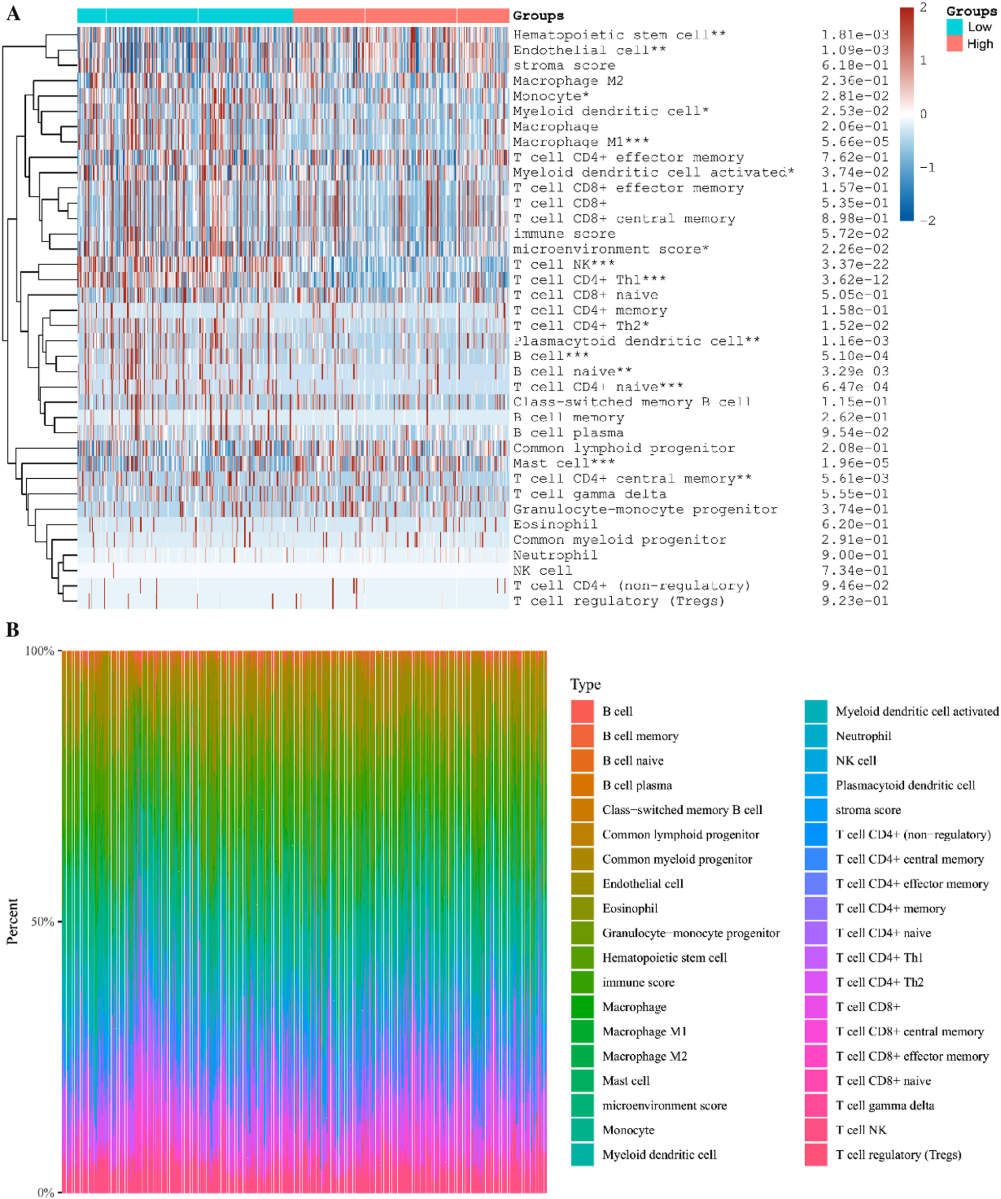
Differential proportions of infiltrating immune cells in kidney renal clear cell carcinoma (KIRC) tissues from patients with low or high expression levels of KL (**A**). The percentage abundance of infiltrating immune cells in renal clear cell carcinoma, with different colors representing different types of immune cells, horizontal axis representing samples, and vertical axis representing the percentage of immune cell content in a single sample (**B**). G1 and G2 are low and high expression groups, respectively. *p < 0.05, **p < 0.01, ***p < 0.001

In Fig. 6, a Spearman correlation analysis was conducted to assess the relationship between the prognosis model score and immune score. The findings indicate that the KL gene has a direct impact on the levels of different immune infiltrating cells in tissues, including B cells, macrophages M1 cells, NK cells, Neutrophils, among others. Using the TCGA-KIRC database, the KIRC population was classified into two groups, namely, the low and high expression groups of KL gene (G1 and G2, respectively), and the correlation between KL gene and the amount of immune infiltrating cells was evaluated. This analysis revealed that the KL gene affects the levels of various immune infiltrating cells to varying degrees in the body (Fig. 7), and that the content of B cell plasma, T cell CD4 + naive, and macrophage M2 in the tissue can influence the level of KL gene. It is postulated that these cells may impact the progression of KIRC.

### Correlation between the expression of immune checkpoint in KIRC tissues and KL gene

**Fig. 8:**
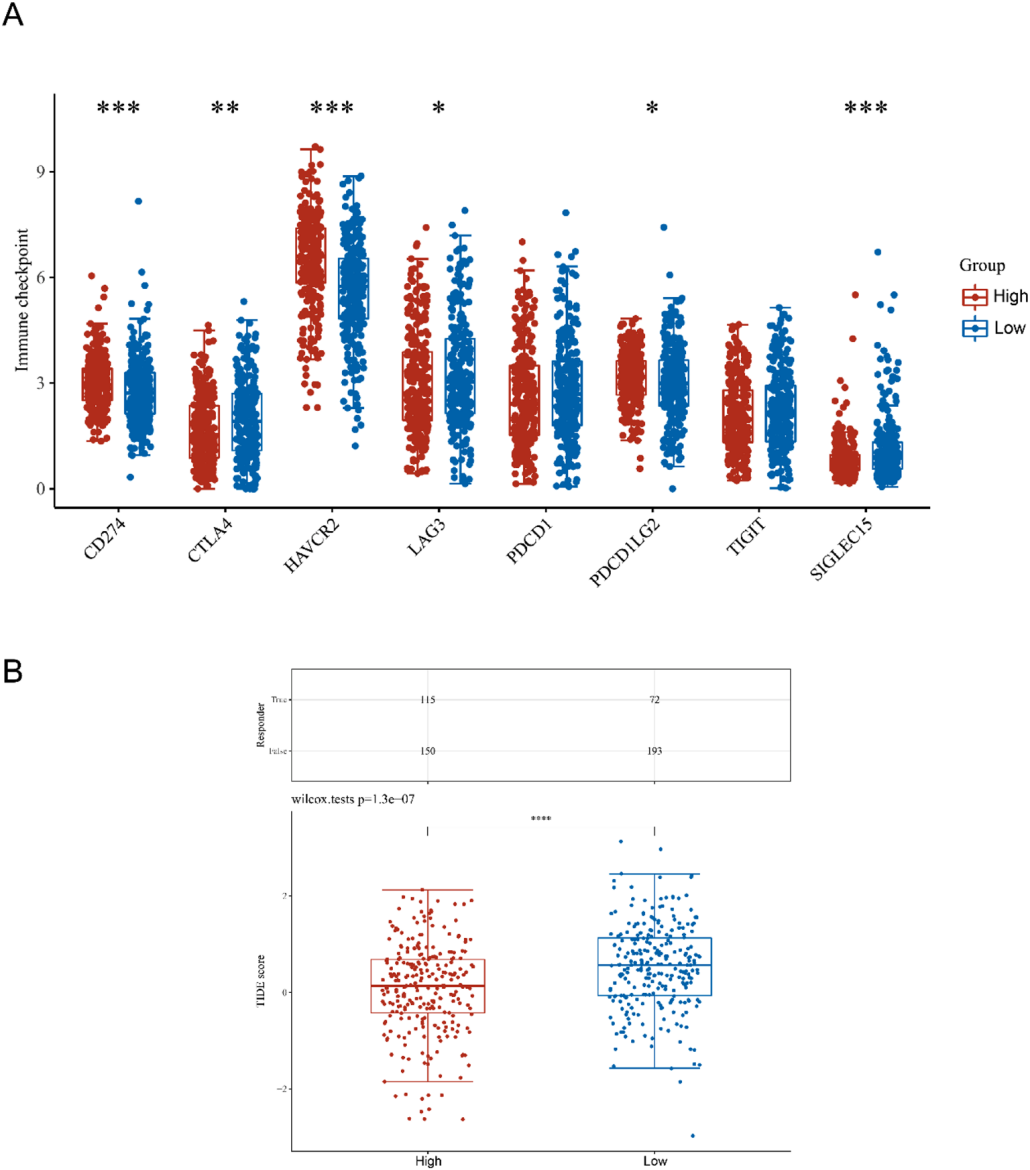
The expression levels of immune checkpoint genes in kidney renal clear cell carcinoma (KIRC) tissues from patients with high or low expression levels of KL (**A**). The differential immune checkpoint blockade response in KIRC tissues from patients with high or low expression levels of KL (**B**). G1 and G2 are high and low expression groups, respectively. *p < 0.05, **p < 0.01, ***p < 0.001

This article mainly discusses the application value of KIRC targeted drugs. In this process, it is also necessary to determine whether the immune checkpoints in KIRC tissue will affect the expression level of necrosis related DEGs. We found that CD274, CTLA4, etc., are closely related to KL (Fig. 8A). This means that KL gene may be sensitive immune checkpoints during KIRC intervention.

This paper also introduces the classic exclusion (TIDE) algorithm to predict the sensitivity of different levels of KL gene and other indicators in tissues to immune checkpoint inhibitors (Fig. 8B). After a series of statistical analysis, it is concluded that the p value of KL gene is less than 0.05, which means that immune checkpoint inhibitors can treat KIRC and have positive significance in improving the survival rate of patients.

### Correlation between microsatellite instability and expression of KL gene

**Fig. 9:**
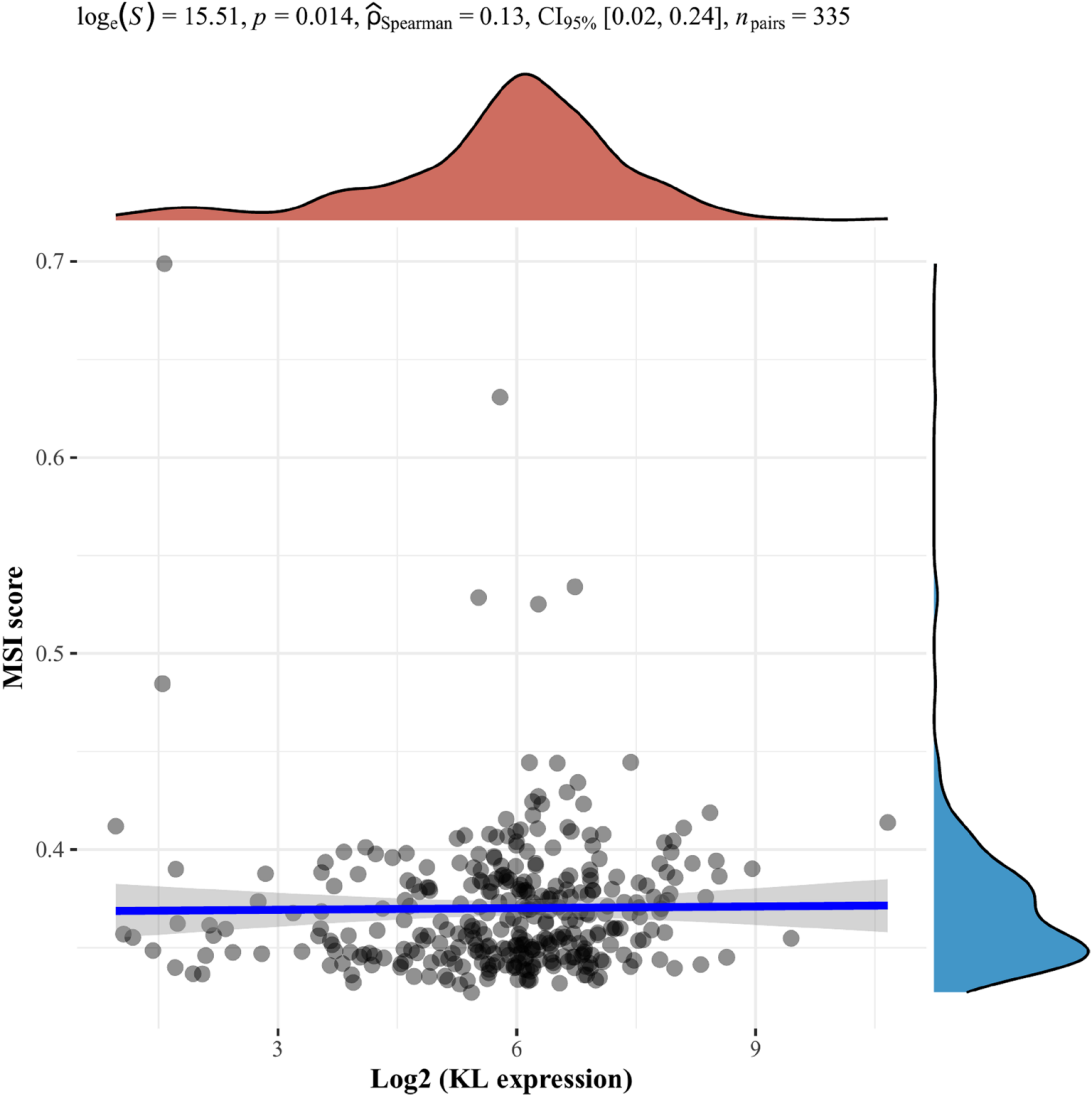
Correlation of microsatellite instability (MSI) with KL expression levels evaluated using Spearman’s correlation analysis. The horizontal axis in the figure represents gene expression distribution, while the vertical axis represents MSI score distribution

To further investigate the relationship between microsatellite instability (MSI) and KL, Spearman correlation analysis of MSI and KL (Fig. 9) was performed. The results revealed that MSI score was significantly correlated with expression of KL (*p* = 0.014).

### Validation of the expression of KL gene in clinical tissue samples

**Fig. 10:**
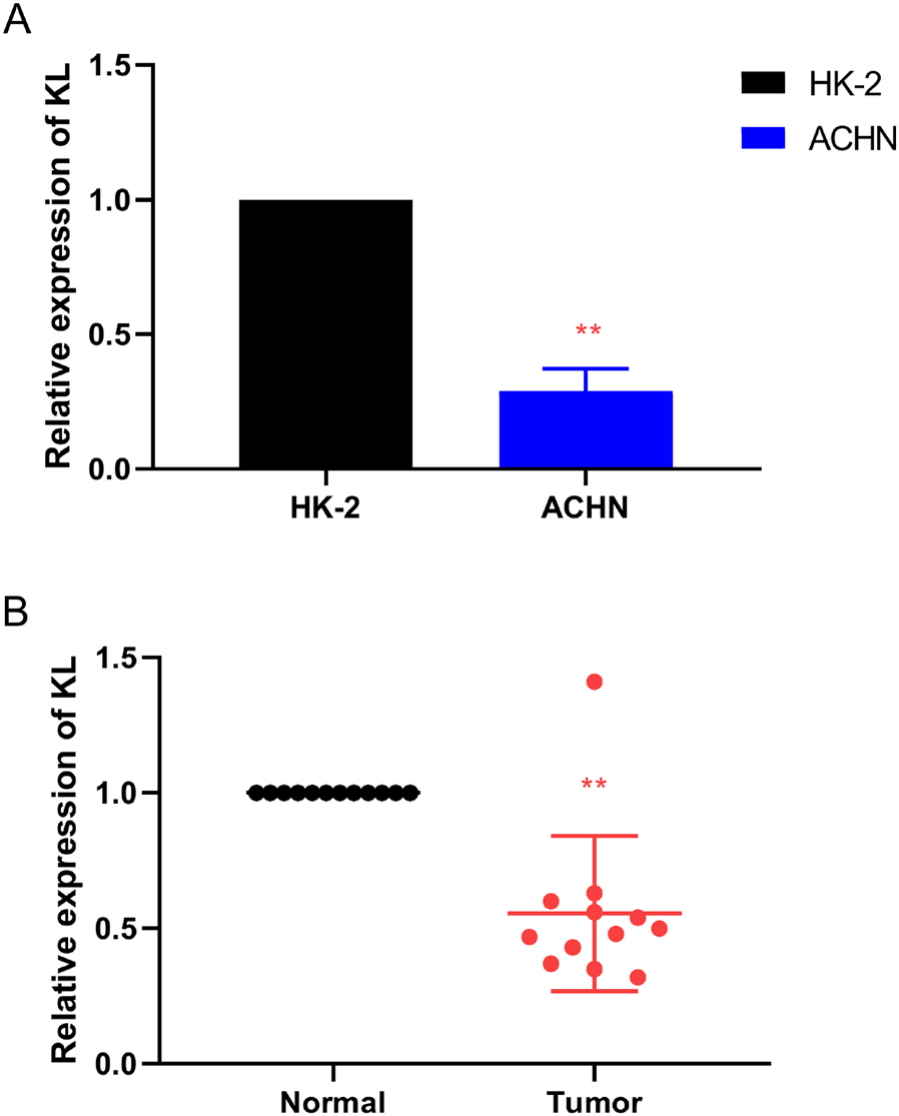
The expression levels of KL in human kidney renal clear cell carcinoma (KIRC) specimens, adjacent healthy tissues, and cell lines (**A**, **B**). Quantitative real-time polymerase chain reaction (qRT-PCR) analysis of KL (**A**) level in KIRC cell lines. GAPDH was used as a housekeeping gene. **B** qRT-PCR analysis of KL levels in paired KIRC tissues (*n* = 12). *p < 0.05, **p < 0.01, ***p < 0.001

**Fig. 11:**
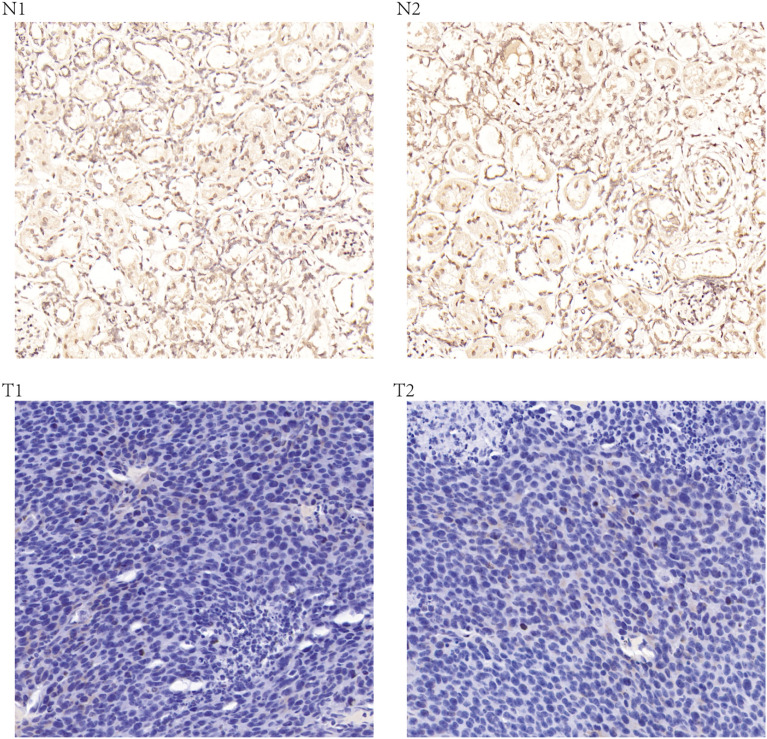
Representative images of KL protein immunochemistry in normal renal tissues and ccRCC tissues. N1 and N2 is the expression of KL in normal renal tissues, T1 and T2 is the expression of KL in ccRCC tissues

In order to determine the actual content of KL gene in KIRC, researchers implemented qRT-PCR in KIRC cell samples. The specific content of the KL gene in the normal kidney cell line (HK-2 cell) was obtained. According to the comparative analysis between groups, the content of KL gene in KIRC cells is less than that in normal kidney cells in Fig. 10A. Further analysis showed that the expression levels of KL gene was less in the pathological tissues (Fig. 10B). IHC method is also introduced to determine the actual content of KL protein in tissues. We found that compared with normal kidney tissue, the content of KL gene in the pathological tissue is less (Fig. 11).

## Discussion

Renal cell carcinoma (RCC) is a common highly malignant tumor of urinary tract, accounting for approximately 90% of renal malignancies, 3% of new cancers in the world. In 2022, it is estimated that about 79,000 people will be diagnosed with RCC in the United States, and about 13,920 people will die from it [12]. About 65% of RCC patients are localized tumors that can usually be successfully controlled by surgery [13], and about 30–70% of patients may develop tumor recurrence and metastasis after surgery. In addition, 30% of RCC patients are found to have metastasized at diagnosis [14]. Most patients can only use palliative treatment, with poor prognosis and 5-year survival rate less than 10% [15–23]. Therefore, actively looking for sensitive markers of renal cell carcinoma plays an important role in the diagnosis of renal cell carcinoma, which has always been the focus of renal cell carcinoma research. At present, the prognosis of KIRC is poor, the disease is difficult to effectively control in a short time, and the probability of tumor recurrence is high. From the current situation, the medical community has not yet identified the sensitive biomarkers of KIRC. The research team hopes to find effective targeted drugs through the mechanism of KIRC, in order to improve the quality of life of patients.

By the Gene GEO database, the GSE168845 dataset was selected for subsequent analyses. The volcano map visually shows 1288 upregulated DEGs selected by the research team, and 1809 downregulated DEGs. In this link, 1793 human IRGs and and 368 ARGs were also objectively reflected by Venn map, and on this basis, 17 co-expression genes were further identified: TNFRSF1B, GBP2, FCGR2B, LIMSI, INPP5D, PTH1R, CALCA, CAT, LRP1, KL, ARG2, PML, MPO, HAMP, HCST, ITGB2 and MAPK1. In this study, we utilized Venn diagram analysis to identify 17 co-expressed immune-aging related genes (IAR-DEGs) from GSE168845, import database, and MSigDB database. GO and KEGG analysis revealed that the functions of these genes were primarily related to “aging”. Subsequently, univariate and multivariate Cox analysis validated 17 genes as potential prognostic factors. However, only KL gene qualified as an independent prognostic factor for ccRCC. We verified the expression of KL gene in tumors and found that it was abnormally low in ccRCC tissues according to the public TCGA database. Additionally, low KL gene expression was negatively correlated with T stage, M stage, pathological stage, and histologic grade (*p* < 0.05). This suggested that decreased KL expression could indicate disease deterioration and shortened overall survival, which was consistent with previous studies [24]. In addition, the nomogram and calibration curves demonstrated effective prediction. Through GSEA analysis, immune and aging-related pathways were found to be mediated by the KL gene. The correlation of KL gene with immune cell infiltration, immune checkpoint, MSI, and TIED score was evaluated where a significant correlation was observed in the expression level of KL gene with immune infiltration, MSI, and TIED score. Based on these findings, KL may have a crucial role in immune cell infiltration in ccRCC and has potential as a new target for immunotherapy. Further analysis of KL mRNA expression in HK-2 human renal normal cell line and ACHN renal cancer cell line showed significantly decreased expression in renal cancer cells, and even lower expression levels of KL mRNA were found in ccRCC tissue compared to adjacent normal kidney tissue. KL protein expression was found to be significantly lower in ccRCC compared to adjacent normal tissues through immunohistochemistry analysis. This observation was supported by consistent KL mRNA and protein expression in ccRCC tissues with clinical data. These results suggest that KL may play a role in the development and progression of renal cell carcinoma.

The role of radiogenomics in renal cancer and in its most common subtype, clear cell renal cancer, is appealing and promising. Considering that the evaluation of a renal lesion is routinely based on CT or MRI images, the possibility of characterizing a potentially malignant lesion in terms of genetic, epigenetic, and pathologic heterogeneity via a non-invasive methodology is an undoubted advantage. Radiogenomics has elevated the interest of many disciplines in the medical sciences due to the possibility to correlate imaging with KL gene, aiming to reach the concept of tailored and personalized medicine [25].

To sum up, there is a correlation between the low expression of KL in ccRCC and patient prognosis and immune infiltration. As a result, the KL gene could potentially serve as a biomarker for ccRCC early diagnosis and prognosis assessment. This discovery may offer a novel approach to targeted and immune therapy for renal cell carcinoma.

## Conclusion

This study utilized bioinformatics to identify immune-aging related genes with significant prognostic value and established a prognostic risk model. Results revealed a robust association between the KL gene and immune score, ICP, and OCLR score, suggesting its potential as a promising target for immunotherapy related to aging in KIRC.

## Data Availability

Publicly available datasets were analyzed in this study. This data can be found here: https://tcga.xenahubs.net

## References

[1] Ljungberg B, Albiges L, Abu-Ghanem Y (2022). European association of urology guidelines on renal cell carcinoma: the 2022 update. Eur Urol.

[2] Siegel RL, Miller KD, Jemal A (2020). Cancer statistics, 2020. CA Cancer J Clin.

[3] Moch H, Cubilla AL, Humphrey PA (2016). The 2016 WHO classification of tumours of the urinary system and male genital organs-part a: renal, penile, and testicular tumours. Eur Urol.

[4] Impfer A, Glass Ä, Zettl H (2019). Renal cell carcinoma diagnosis and prognosis within the context of the WHO classification 2016. Urologe A.

[5] Spadaccino F, Gigante M, Netti GS (2021). The ambivalent role of miRNAs in carcinogenesis: involvement in renal cell carcinoma and their clinical applications. Pharmaceuticals.

[6] Romeo A, Garcia Marchiñena P, Jurado AM (2020). Renal fossa recurrence after radical nephrectomy: current management and oncological outcomes. Urol Oncol.

[7] Go H, Kang MJ, Kim PJ (2019). Development of response classifier for vascular endothelial growth factor receptor(VEGFR)-tyrosine kinase inhibitor(TKI) in metastatic renal cell carcinoma. Pathol Oncol Res.

[8] Kuro-o M, Matsumura Y, Aizawa H (1997). Mutation of the mouse klotho gene leads to a syndrome resembling ageing. Nature.

[9] Hanahan D, Weinberg RA (2011). Hallmarks of cancer: the next generation. Cell.

[10] Zhou X, Wang X (2015). Klotho: a novel biomarker for cancer. J Cancer Res Clin Oncol.

[11] Zhu Y, Xu L, Zhang J (2013). Klotho suppresses tumor progression via inhibiting PI3K/Akt/GSK3β/Snail signaling in renal cell carcinoma. Cancer Sci.

[12] Ikushima M, Rakugi H, Ishikawa K (2006). Anti-apoptotic and anti-senescence effects of Klotho on vascular endothelial cells. Biochem Biophys Res Commun.

[13] Arabsalmani M, Mohammadian-Hafshejani A, Ghoncheh M (2017). Incidence and mortality of kidney cancers, and human development index in Asia; a matter of concern[J]. J Nephropathol.

[14] Kabaria R, Klaassen Z, Terris MK (2016). Renal cell carcinoma: links and risks[J]. Int J Nephrol Renovasc Dis.

[15] Siegel R, Ma J, Zou Z (2014). Cancer statistics, 2014. CA Cancer J Clin.

[16] Siegel RL, Miller KD, Fuchs HE (2022). Cancer statistics, 2022. CA Cancer J Clin.

[17] Kathuria-Prakash N, Drolen C, Hannigan CA (2021). Immunotherapy and metastatic renal cell carcinoma: a review of new treatment approaches. Life.

[18] Kroeger N, Choueiri TK, Lee JL (2014). Survival outcome and treatment response of patients with late relapse from renal cell carcinoma in the era of targeted therapy. Eur Urol.

[19] Kidney and Renal Pelvis Cancer [EB/OL]. https://seer.cancer.gov/statfacts/html/kidrp.html. Accessed 08 June 2022.

[20] Pandey N, Lanke V, Vinod PK (2020). Network-based metabolic characterization of renal cell carcinoma. Sci Rep.

[21] Fu Q (2016). Positive intratumoral chemokine (C–C motif) receptor 8 expression predicts high recurrence risk of post-operation clear-cell renal cell carcinoma patients. Oncotarget.

[22] Barata PC, Rini BI (2017). Treatment of renal cell carcinoma: current status and future directions. CA Cancer J Clin.

[23] Senbabaoglu Y (2016). Tumor immune microenvironment characterization in clear cell renal cell carcinoma identifies prognostic and immunotherapeutically relevant messenger RNA signatures. Genome Biol.

[24] Dizman N, Arslan ZE, Feng M, Pal SK (2020). Sequencing therapies for metastatic renal cell carcinoma. Urol Clin North Am.

[25] Ferro M, Musi G, Marchioni M (2023). Radiogenomics in renal cancer management-current evidence and future prospects. Int J Mol Sci.

